# Comparison of long and short axis quantification of left ventricular volume parameters by cardiovascular magnetic resonance, with ex-vivo validation

**DOI:** 10.1186/1532-429X-13-40

**Published:** 2011-08-11

**Authors:** Helene Childs, Lucia Ma, Michael Ma, James Clarke, Myra Cocker, Jordin Green, Oliver Strohm, Matthias G Friedrich

**Affiliations:** 1 Stephenson Cardiovascular MR Centre at the Libin Cardiovascular Institute of Alberta, Departments of Cardiac Sciences and Radiology, University of Calgary, AB, Canada; 2 Dept. of Nuclear Medicine and Radiology, Dalhousie University, Halifax, NC, Canada; 3 Siemens Healthcare, Henkestr. 12791054 Erlangen, Germany

## Abstract

**Background:**

The purpose of the study was to compare the accuracy and evaluation time of quantifying left ventricular (LV), left atrial (LA) volume and LV mass using short axis (SAX) and long axis (LAX) methods when using cardiovascular magnetic resonance (CMR).

**Materials and methods:**

We studied 12 explanted canine hearts and 46 patients referred for CMR (29 male, age 47 ± 18 years) in a clinical 1.5 T CMR system, using standard cine sequences. In standard short axis stacks of various slice thickness values in dogs and 8 mm slice thickness (gap 2 mm) in patients, we measured LV volumes using reference slices in a perpendicular, long axis orientation using certified software. Volumes and mass were also measured in six radial long axis (LAX) views.

LV parameters were also assessed for intra- and inter-observer variability. In 24 patients, we also analyzed reproducibility and evaluation time of two very experienced (> 10 years of CMR reading) readers for SAX and LAX.

**Results:**

In the explanted dog hearts, there was excellent agreement between ex vivo data and LV mass and volume data as measured by all methods for both, LAX (r^2 ^= 0.98) and SAX (r^2 ^= 0.88 to 0.98). LA volumes, however, were underestimated by 13% using the LAX views. In patients, there was a good correlation between all three assessed methods (r^2 ^≥ 0.95 for all). In experienced clinical readers, left-ventricular volumes and ejection fraction as measured in LAX views showed a better inter-observer reproducibility and a 27% shorter evaluation time.

**Conclusion:**

When compared to an ex vivo standard, both, short axis and long axis techniques are highly accurate for the quantification of left ventricular volumes and mass. In clinical settings, however, the long axis approach may be more reproducible and more time-efficient. Therefore, the rotational long axis approach is a viable alternative for the clinical assessment of cardiac volumes, function and mass.

## Background

Cardiovascular magnetic resonance imaging (CMR) is well recognized as a gold standard for functional imaging and assessment. CMR using state-of-the-art sequences has been shown to be very reproducible, is considered appropriate for many clinical indications [1] and there is consensus about its clinical use and methodology [2]; newer sequences, however, have not been evaluated against an ex vivo gold standard. Furthermore, short axis (SAX) stacks, considered the standard approach for quantifying left ventricular (LV) volumes and function and used as such for validation [3], are time consuming and there are partial volume effects leading to problems that arise upon defining the slice area in the most basal and most apical slices [4]. Inclusion or exclusion of basal slices is of particular interest since until now, the blood volume in the LV outflow tract is often excluded, while atrial volume may be erroneously included. Likely due to this problem, the utilization of SAX views may be hampered by a lesser reproducibility [5].

Long axis views (LAX) have been proposed as an alternative to SAX-derived methods [6]. We aimed to validate state-of-the-art sequences in SAX and rotational LAX views in explanted canine hearts and to compare the two methods in clinical CMR studies. This is of clinical importance for LV function and mass analysis, but also for correlating these results with tissue abnormalities such as high signal intensity areas in late Gd enhancement or T2-weighted images.

## Materials and methods

All examinations were performed on a 1.5 T system (MAGNETOM Avanto^®^, Siemens Healthcare, Erlangen, Germany). All quantitative CMR parameters were assessed using certified software (cmr^42^, Circle Cardiovascular Imaging Inc., Calgary, Canada). All readers had training on drawing contours with special emphasis on how to avoid misinterpretation of the basal slice.

### Ex vivo studies

**Figure 1:**
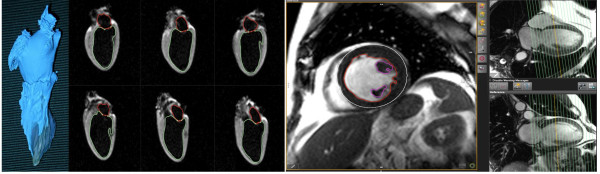
**Mold of explanted dog heart and long axis CMR images**. Left: Mold of the left ventricle and left atrium of an explanted dog heart. Right: Six ex vivo CMR long axis views of the mold-filled heart. Contours are shown for the subendocardial border of the left atrium and left ventricle.

Twelve freshly explanted canine hearts were filled with MRI-compatible, autopolymerizing acrylic resin (Dentsply, Caulk, York, PA, USA) for an accurate representation of cavities, and then imaged with both radial LAX and SAX orientations for comparison. Three contiguous SAX stacks with full LA and LV coverage were analyzed separately (slice thickness: 10 mm, 8 mm, and 5 mm) and six radial LAX slices were obtained as per published protocols [5]. CMR dimensions were assessed (Figure 1) in a blinded fashion and results were compared to mold data. Molds were excised and volumes were determined by, displacement, a digital scale was used to weigh LV myocardium.

### Subjects

We assessed 46 individuals, (29 male, 17 female; mean age 47 ± 18, range 15-79 years). 4 were healthy volunteers without evidence for heart disease, while 42 patients were randomly selected from clinical referrals for functional imaging, tissue characterisation and/or viability. The study was approved by the local ethics committee and informed consent was obtained from each individual.

### CMR sequence

Standard Steady-State-Free-Precession (SSFP) cine sequences were used for all approaches. Typical parameters were: TE 1.1 ms, TR 2.31 ms, FA 75°, matrix 340 × 284 mm, pixel size 1.3 mm × 1.4 mm and an IPAT factor 2. With 25 lines per segment, the effective repetition time typically was 57 ms. SAX acquisition was performed as multiple short axes across the entire LV in an imaging plane perpendicular to the LAX of the LV with a slice thickness of 8 mm and a 2 mm gap. The LAX slices were performed using 6 slices rotating in 30 degree increments around the anatomical LV LAX.

### Image Analysis

CMR image analysis was performed using certified software (cmr^42^, Circle Cardiovascular Imaging Inc., Calgary, Canada) by well-trained observers. Endocardial and epicardial contours were drawn manually for the LV at end-systole and end-diastole in each data set.

**Figure 2:**
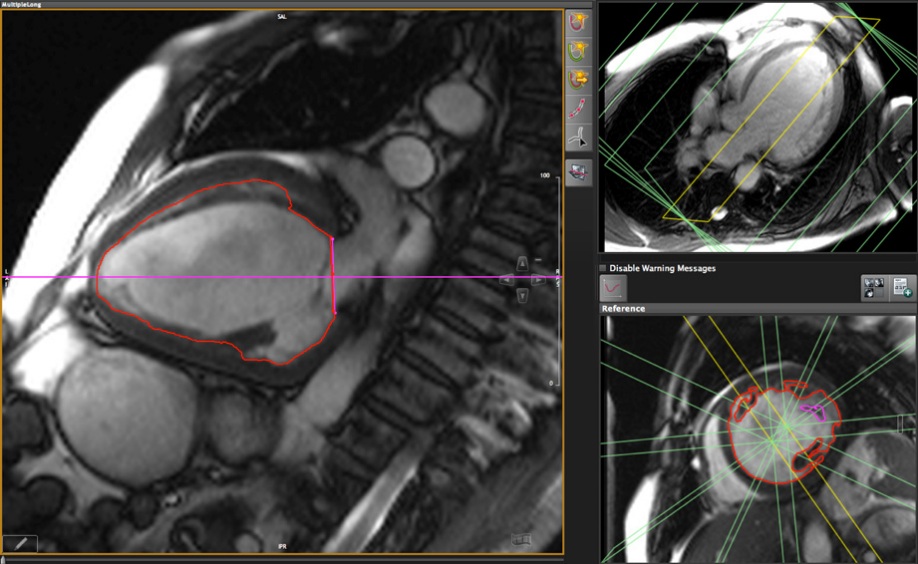
**LV function analysis in short axis images using long axis views as a cross-reference**. Left panel: Diastolic mid-ventricular short axis view with contours. Right upper panel: Example long axis reference view in a 4-chamber orientation. Right lower panel: Example long axis reference view in a 2-chamber orientation. The orange lines represent the location of the cross-sectional long axis views used for measurements.

**Figure 3:**
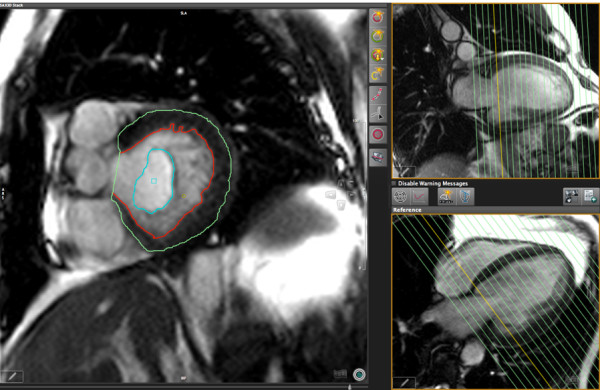
**LV function analysis in long axis images using short axis views as a cross-reference**. Left panel: Diastolic long axis view with endocardial contour. Right upper panel: Example short axis reference view in a 3-chamber orientation. Right lower panel: Example short axis reference view in short axis orientation. The orange lines represent the location of the cross-sectional views used for measurements.

In contrast to an uncontrolled approach for identifying the most basal short axis slice by identifying the most basal image which contains at least 50% of circumferential myocardium [7], we used perpendicular images with lines representing the position of the basal and the apical short axis planes as automatically provided by the software to control for the position and decide on the inclusion of this slice (Figure 2). The blood volume encompassed by the mitral valve was excluded while LV outflow tract volume was included as determined by the LAX cross-reference. Papillary muscles and trabeculations were included in LV mass and volume calculations. The interventricular septum was included in the LV mass. As LAX cross-reference views, 3- and 4-chamber views were typically used (Figure 3).

**Figure 4:**
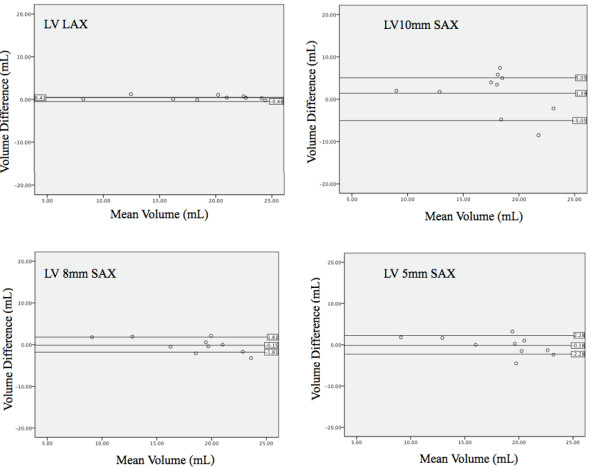
**Correction of atrial volume next to the mitral valve**. Left panel: Diastolic basal short axis view with contours. The green contour encircles the atrial portion of the volume in this slice and is excluded from the ventricular volume. Right upper panel: Example long axis reference view in a 2-chamber orientation. Right lower panel: Example long axis reference view in a 4-chamber orientation. The orange lines represent the location of the cross-sectional long axis views used for measurements.

As for SAX views, endocardial and epicardial contours were manually drawn in end-systolic and end-diastolic frames of all LAX views. Contours excluded trabeculations while including most of the papillary muscles. The blood volume in the mitral valve was excluded with inclusion of blood volume in the LV outflow tract up to the aortic valve (Figure 4).

We assessed intra-observer variability by having one observer analyzing volumes with SAX twice, while a different observer analyzed volumes with LAX approach twice. To minimize observer bias, each of the two less experienced readers read only one of the orientations, either SAX or LAX. This may have introduced a bias for the time needed. Both readers, however, had similar training before the study. Furthermore, for experienced readers such a bias can be excluded, since they read both, SAX and LAX view in separate sessions.

For all methods, end-diastolic volume (EDV), end-systolic (ESV), stroke volume (SV), ejection fraction (EF), and LV mass were assessed by three independent observers. LV mass was measured at end-systole by both methods because of the easier definition of the endocardial borders in the contracted myocardium. The time needed to complete assessments was recorded in a randomly selected subgroup of 12 individuals.

### Statistical Analysis

**Figure 5:**
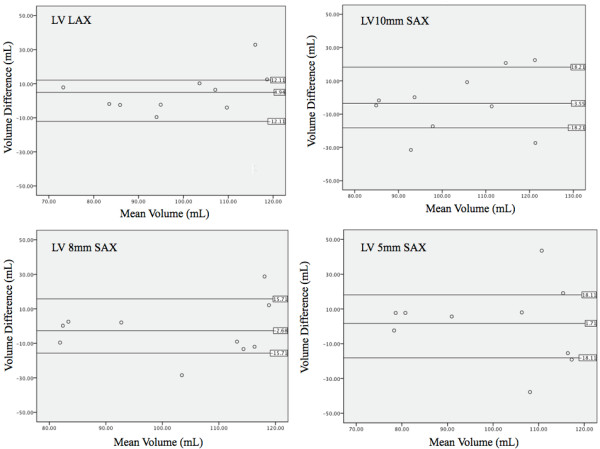
**Bland-Altman analysis of left ventricular volume**. Overall bias and 95% limits of agreement between mold data and different methods for LV volume determination. Standard compared to CMR measurements using radial (top left), short axis 10 mm (top right), short axis 8 mm (bottom left), and short axis 5 mm (bottom right) techniques. The long axis method showed the least amount of variation and was on average closest to the real volume.

**Figure 6:**
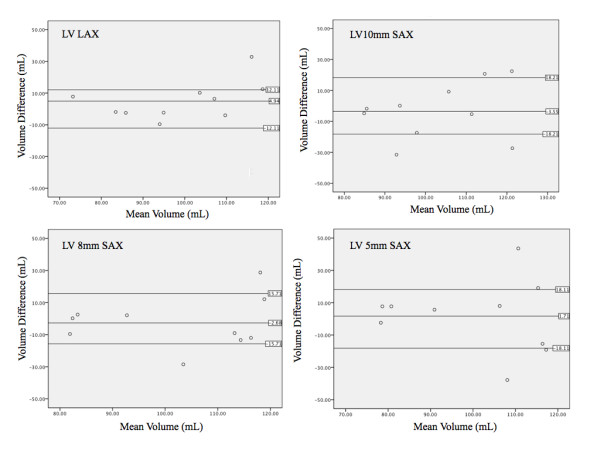
**Bland-Altman analysis of left ventricular mass**. Overall bias and 95% limits of agreement between mold data and different methods for LV volume determination. Standard compared to CMR measurements using radial (top left), short axis 10 mm (top right), short axis 8 mm (bottom left), and short axis 5 mm (bottom right) techniques.

**Figure 7:**
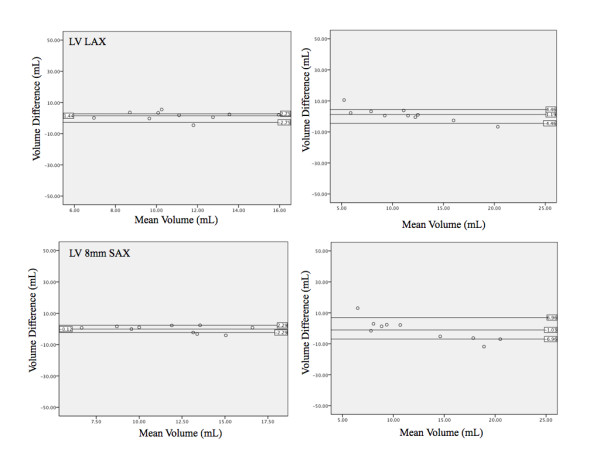
**Bland-Altman analysis of left atrial volume**. Overall bias and 95% limits of agreement between mold data and different methods for LV volume determination. Standard compared to CMR measurements using radial (top left), short axis 10 mm (top right), short axis 8 mm (bottom left), and short axis 5 mm (bottom right) techniques.

CMR data was compared to mold measurements using a paired t test and Pearson's correlation coefficient. Inter- and intra-observer variability was analyzed using linear regression to calculate the square of the correlation coefficient, (r^2^). The mean difference between observations was calculated by averaging the absolute value of the difference between methods. Likewise, the difference for inter- and intra-observer variability was calculated as the absolute value of the difference divided by the initial measurement. Both absolute and relative differences between methods were calculated and Bland-Altman plots calculated (Figure 5, 6 and 7). All calculations were completed using statistics software (Microsoft Excel 12.2.6 for Mac, Microsoft Corporation, USA).

## Results

### Explanted canine hearts

LV results obtained from SAX and LAX views were in excellent agreement with the results from the molds (Pearson's correlation coefficient, r ≥ 0.6, p < 0.05). LA volumes were significantly correlated with SAX methods (r ≥ 0.6, p < 0.05) (table 1). There was no significant difference between LAX and SAX techniques for measurements of LV volumes, LA volumes or LV mass (p > 0.05). There was also no significant difference between CMR-derived measurements and actual values of LV mass (explanted hearts) and LV volumes (mold).

**Table 1:** Differences between CMR measurements and actual results with correlation coefficients in explanted canine hearts

	Reference (mold)	LAX	SAX 10 mm	SAX 8 mm	SAX 5 mm
**LV Volume**	23.3 ± 5.3 ml	-2.5 ± 1.1% CV = 1.18	-7.0 ± 7.8% CV = 3.63	-0.7 ± 4.6% CV = -12.1	+0.3 ± 5.3% CV = -12.7

**LA Volume**	11.8 ± 7.5 ml	-13.3 ± 7.0% CV = 1.61	-4.2 ± 6.9% CV = 3.49	-3.3 ± 6.9% CV = 6.07	+5.3 ± 9.1% CV = -9.24

**LV Mass**	99.6 ± 5.5 g	-3.3 ± 3.0% CV = 2.68	+7.5 ± 5.6% CV = -3.49	+4.0 ± 4.2% CV = -5.31	+0.1 ± 6.0% CV = 17.3

Difference CV shows the degree of variation by providing the ratio of the standard deviation relative to the mean. Values are expressed as mean ± SE. LV: Left ventricular; LA: Left atrial; LAX: Long axis method; SAX: Short axis method; CV: coefficient of variation of the difference between mold and CMR values.

### Patients

Seventeen of the 42 patients referred for CMR did not reveal any abnormalities, while 25 had various cardiac conditions. Image quality was good in all cases, none of the patients were excluded from the analysis.

There was a good correlation with no significant difference between SAX and LAX evaluation (Table 2).

**Table 2:** Volumetric results as calculated from the different analysis methods in patients

	Short axis views	Long axis views
**LVEDV**	177 ± 99 ml	182 ± 101 ml

**LVESV**	94 ± 102 ml	91 ± 97 ml

**LV-EF**	55 ± 16%	55 ± 15%

**LV mass**	144 ± 64 g	141 ± 62 g

None of the differences was statistically significant.

Inter-observer variability was smaller with LAX measurements for LVEDV, LVESV and LV-EF (Table 3). Results of LV mass quantification did not differ between observers (p = NS).

**Table 3:** Absolute inter-observer differences (%) using different acquisition orientations in patients.

	Short axis views	Long axis views
**LVEDV**	11.2 ± 7.9 ml	4.4 ± 4.6 ml *

**LVESV**	18.6 ± 12.5 ml	7.0 ± 5.1 ml *

**LV-EF**	7.2 ± 5.7%	3.0 ± 1.7%*

**LV mass**	14.0 ± 14.4 g	7.1 ± 6.8 g

*: p < 0.05 vs. standard and cross-referenced short axis views. Differences between standard and cross-referenced short axis view measurements were not significant.

For less experienced readers, the time of evaluation for SAX was 13 minutes and 8 seconds, while the time of evaluation for LAX was slightly less, 12 minutes and 42 seconds. Required evaluation times for experienced readers are shown in table 4.

**Table 4:** Time needed for evaluation using different acquisition orientations in patients.

	Short axis views	Long axis views	Time saving
**Experienced reader**	8:42 ± 4:38	6:24 ± 0:49*	26%

**Less experienced reader**	13:08 ± 7:14	12:42 ± 3:34	3%

*: p < 0.05 vs. standard and cross-referenced short axis views.

## Discussion

Our data indicate that both, cross-referenced short axis and long axis views provide accurate measurements of volumetric LV parameters. This is not specific for CMR and has implications for other imaging techniques such as CT. Furthermore, our data indicate that long axis views take less time for evaluation. Another advantage of long axis views is the low susceptibility to errors induced by misinterpretation of slices close to the mitral valve. In fact, a post-hoc analysis of our data showed that errors of standard short axis data were due to inclusion or exclusion of the most basal slice, while there was no difference between cross-referenced and long axis results. Therefore, long axis planes may be a useful approach in most routine clinical situations.

LV mass was larger when calculated by SAX. This could be due to the inclusion of trabeculations and papillary muscles in SAX while only papillary muscles were included in LAX; however, we were concerned that including both trabeculations and papillary muscles in the LAX would lead to an over-estimation in LV mass due to the use of rotational volumetry calculation [5]. Another possible explanation is that SAX images experience partial volume effects in basal and apical slices while in radial LAX images the identification of subendocardial borders is easier.

Most validation studies have been performed using SAX measurements of LV mass [8-10]. Furthermore, tissue characterization often is performed in SAX views and therefore this view may be better for correlating results between sequences. Since abnormalities seen in images used for tissue characterization should be verified by cross-sectional views and therefore both long and short axis views are required anyway, a comparison of tissue characteristics with the cine images is equally possible. More recent studies indicate that long-axis based methods may be equally accurate yet may have a smaller intra-observer variability [6]. This may be due to the clear visualisation of both mitral and aortic valve planes and the reduction of partial volume effects allowing for well-defined myocardial boundaries [5]. So, while LAX methods use geometric assumptions, the advantage of having a perpendicular view on the basal and apical borders of the left ventricle may outweigh the disadvantage of partially replacing actual measurements by computed data.

### Limitations

We did not systematically assess the impact of ventricular shape criteria on our results. Although this has not been studied systematically, a highly irregular morphology may limit the accuracy of LAX data [11]. In the LAX views, traceless and papillary muscles were only partially included into LV mass, whereas they were completely included in the SAX stack evaluation. This is due to the fact, that inclusion of small structures (i.e. smaller than the distance tracked by the 30° rotation between LAX planes) leads to overestimation of the volume of these tissues (unpublished data). This inconsistency between the methods however was not associated with significant differences between methods; yet, it may explain the non-significant trend toward underestimation of LV mass by LAX. For clinical scenarios, the lack of significant differences let this problem appear irrelevant. In the SAX method, we used no gaps for the dog studies, but had gaps in patients. The reason for using no gaps in dogs was simply to compare the results by both methods excluding other confounders. Resembling clinical scenarios, however, we applied gaps in patient studies as typically done in clinical applications and clinical research [12]. The applicability of our results to other readers may be limited by individual approaches how to deal with inclusion or exclusion of papillary muscles and trabecular tissue.

## Conclusion

When compared to an ex vivo standard, both, short axis and long axis techniques are highly accurate for the quantification of left ventricular volumes and mass. In patients, however, the long axis approach may be more reproducible and more time-efficient. For the analysis of left ventricular volumes by tomographic techniques, mass and function in hearts without severe shape alterations, a long axis approach may be a viable alternative

## Competing interests

Oliver Strohm is an advisor to Circle CV Imaging Inc., Calgary, Canada. and Matthias G. Friedrich is on the board of directors of Circle CV Imaging Inc., Calgary, Canada.

## Authors' contributions

LM, MM, MC, and JC were involved with the patient component of the study and assisted in drafting the manuscript. HC completed the ex-vivo component of the study and assisted in drafting the manuscript. JG was involved in both patient and ex-vivo scanning components.

MF and OS designed and coordinated this study. All authors read and approved the final manuscript.
